# The Dutch Parelsnoer Institute - Neurodegenerative diseases; methods, design and baseline results

**DOI:** 10.1186/s12883-014-0254-4

**Published:** 2014-12-31

**Authors:** Pauline Aalten, Inez HGB Ramakers, Geert Jan Biessels, Peter Paul de Deyn, Huiberdina L Koek, Marcel GM OldeRikkert, Ania M Oleksik, Edo Richard, Lieke L Smits, John C van Swieten, Laura K Teune, Aad van der Lugt, Frederik Barkhof, Charlotte E Teunissen, Nico Rozendaal, Frans RJ Verhey, Wiesje M van der Flier

**Affiliations:** Alzheimer Center Limburg, School for Mental Health and Neuroscience (MHeNS), Maastricht University Medical Center, P.O. Box 616, 6200 MD Maastricht, The Netherlands; Departments of Neurology and Geriatrics, University Medical Center Utrecht, Utrecht, The Netherlands; Department of Neurology, Alzheimer Research Cente, University Medical Center Groningen, Utrecht, The Netherlands; Alzheimer Center Nijmegen, Radboud University Medical Center Nijmegen, Nijmegen, The Netherlands; Department of Gerontology and Geriatrics, Leiden University Medical Center, Leiden, The Netherlands; Department of Neurology, Academic Medical Center Amsterdam, Amsterdam, The Netherlands; Alzheimer Center Amsterdam, VU Medical Center Amsterdam, Amsterdam, The Netherlands; Departments of Neurology and Radiology, Erasmus Medical Center, Rotterdam, The Netherlands; Department of Clinical Chemistry, Neurochemistry Laboratory and Biobank, VU Medical Center Amsterdam, Amsterdam, The Netherlands

**Keywords:** Dementia, Alzheimer’s disease, Design, Biobank, Research infrastructure

## Abstract

**Background:**

The *Parelsnoer Institute* is a collaboration between 8 Dutch University Medical Centers in which clinical data and biomaterials from patients suffering from chronic diseases (so called “Pearls”) are collected according to harmonized protocols. The Pearl Neurodegenerative Diseases focuses on the role of biomarkers in the early diagnosis, differential diagnosis and in monitoring the course of neurodegenerative diseases, in particular Alzheimer’s disease.

The objective of this paper is to describe the design and methods of the Pearl Neurodegenerative Diseases, as well as baseline descriptive variables, including their biomarker profile.

**Methods:**

The Pearl Neurodegenerative Diseases is a 3-year follow-up study of patients referred to a memory clinic with cognitive complaints. At baseline, all patients are subjected to a standardized examination, including clinical data and biobank materials, e.g. blood samples, MRI and cerebrospinal fluid. At present, in total more than 1000 patients have been included, of which cerebrospinal fluid and DNA samples are available of 211 and 661 patients, respectively. First descriptives of a subsample of the data (n = 665) shows that patients are diagnosed with dementia (45%), mild cognitive impairment (31%), and subjective memory complaints (24%).

**Discussion:**

The Pearl Neurodegenerative Diseases is an ongoing large network collecting clinical data and biomaterials of more than 1000 patients with cognitive impairments. The project has started with data analyses of the baseline characteristics and biomarkers, which will be the starting point of future specific research questions that can be answered by this unique dataset.

## Background

### The parelsnoer institute

The Parelsnoer Institute (PSI; www.string-of-pearls.org) is a collaboration of the eight Dutch University Medical Centers (UMCs), designed to create an infrastructure for collection of clinical data and biomaterial from patients with chronic diseases. The conceptual starting point for PSI is the idea that clinical practice and research can mutually strengthen each other. By providing a harmonized infrastructure for the clinical evaluation of patients, data collection and storage of data in a centralized database, PSI enables the use of multicenter clinical data to answer research questions targeted to improve patient care. PSI takes advantage of the Dutch context, where it is relatively easy to achieve nationwide coverage of disease populations because of the close connections. Initially, eight patient cohorts were created, symbolized by “Pearls”. Besides Neurodegenerative diseases, in particular dementia, the Pearls include stroke, type 2 diabetes mellitus, hereditary colorectal cancer, inflammatory bowel diseases (Crohn’s disease and ulcerative colitis), leukaemia, renal failure, and rheumatoid arthritis and arthrosis.

### Neurodegenerative diseases

Dementia is one of the major health care challenges of the 21st century. There are currently more than 35 million people affected with dementia worldwide, of which Alzheimer's disease (AD) is the most common type [1]. Other types of dementia include vascular dementia, dementia with Lewy bodies, frontotemporal dementia and more rare variants [2,3]. To date, there is no cure for AD or any of the other types of dementia. In fact, we are only just starting to understand the aetiology and disease course of these diseases. Research in AD has long time been hampered by the fact that neither plaques nor tangles (nor any of the proteins involved in other types of dementia) could be measured during life. The development of biomarker assays as diagnostic tests in cerebrospinal fluid (CSF) and amyloid imaging using PET has been a great breakthrough, together with structural imaging using MRI, acting as a catalyst for AD research. The biomarkers have been used to develop a hypothetical model, which serves to test hypotheses regarding the order of events leading to AD dementia [4,5]. In addition, there is increasing evidence that some biomarkers can be used as early diagnostic markers in the continuum of AD. Results from several large studies have demonstrated that a combination of CSF biomarkers and neuroimaging measures is useful in predicting the likelihood of developing AD [6]. This has resulted in their incorporation in new diagnostic criteria, as a means to provide evidence for the presence of Alzheimer pathology [7-9]. It is now widely recognized that AD has a long pre-dementia stage, as brain changes start to accumulate as early as 15 to 20 years before dementia onset [10]. Mild cognitive impairment (MCI) refers to a stage where memory impairment can be objectified, but the patients’ daily functioning is still intact. In university memory centres, patients with MCI have a mean annual conversion rate of 10% to dementia, mostly AD [11]. With the use of biomarkers, it is possible to make a better prediction of conversion to AD, though still far from perfect [12]. Preclinical AD is the proposed terminology to describe the stage when the earliest brain changes have begun, but there are no clinical signs yet [13].

### Aim pearl neurodegenerative diseases

The aim of the Pearl Neurodegenerative diseases is to provide an infrastructure for the study of biomarkers for early diagnosis predicting clinical decline and incident dementia in those who are not demented yet, and for differential diagnosis and discriminating AD from other etiologies. The evaluation of a broad spectrum of patients, representing a tertiary memory clinic population, allows answering both questions. This is achieved by establishing a biobank containing blood, CSF, DNA with available extensive clinical phenotyping of patients, neuropsychological tests, and brain MRI. The data collection is completed by data from annual follow-up assessments.

In this paper, we describe the design and methods of the Pearl Neurodegenerative diseases as well as baseline descriptive variables. In addition, we extracted DNA and CSF from the biobank, and determined APOE genotype, and CSF concentrations of amyloid-beta 1–42 (ab42), total tau and tau phosphorylated at threonine 181 (ptau).

## Methods

### Study design

PSI is a collaboration of all eight UMCs in the Netherlands (University of Maastricht, Amsterdam (Free University and Amsterdam Medical Center), Rotterdam, Leiden, Groningen, Utrecht, and Nijmegen), and is jointly financed by the Dutch government and the eight Dutch UMCs. The Pearl “Neurodegenerative diseases” is a prospective, multi-center cohort study, focusing on tertiary memory clinic patients with cognitive problems including dementia. The memory clinics are embedded at the department of psychiatry (1 center), neurology (5 centers), or geriatrics (2 centers). Patients are enrolled from March 2009 and initially followed annually for two years. The goal was to include 100 patients per center, resulting in a database of 800 patients.

### Patients

In order to include the whole cognitive spectrum from subjective cognitive complaints to dementia, broad inclusion criteria were chosen. Inclusion criteria included patients referred to a memory clinic for the evaluation of cognitive problems, with a clinical dementia rating scale (CDR) [14] score of 0, 0.5, or 1, and a Mini Mental State Examination (MMSE) [15] of 20 or higher. Exclusion criteria were: Normal Pressure Hydrocephalus, Morbus Huntington, recent Transient Ischemic Attack (TIA) or Cerebrovascular Accident (CVA) (<2 years), TIA/CVA followed by cognitive decline (within three months), history of schizophrenia, bipolar disorder or psychotic symptoms not otherwise specified or previous treatment for these diseases, current major depressive disorder (DSM IV), cognitive problems due to alcohol abuse, brain tumor, epilepsy, encephalitis, mental incompetence for deciding participation, absence of a reliable informant, or the expectation that a follow-up assessment after one year was not possible.

### Baseline minimal dataset

At baseline, each center collected a harmonized minimal dataset, consisting of variables highly relevant for the early diagnosis of neurodegenerative diseases. Data were collected according to Standard Operating Procedures (SOPs) specifically defined for the Pearl “Neurodegenerative diseases”.

The minimal dataset consisted of clinical data and a cognitive assessment. In addition, a MRI of the brain was made and blood samples and CSF samples (optional) were collected for biobanking purposes. Table 1 gives an overview of the data collection per assessment.

**Table 1:** **Flowchart data collection**

	**Baseline**	**1-year FU**	**2-year FU**
Informed consent	X		
Clinical data	X	X	X
Cognitive assessment	X	X	X
Scales and questionnaires	X	X	X
Blood (serum and plasma)	X		X
DNA	X		X
MRI	X		X
CSF (optional)	X		

DNA: Deoxyribonucleic Acid, MRI: Magnet Resonance Imaging, CSF: Cerebrospinal fluid.

**Table 2:** **Basic demographic and clinical variables of subsample**

**N**	**665**
Gender, F	276 (42%)
Age, years	70 ± 10; <65 year: 31%
MMSE	26 ± 3
CDR; 0; 0.5; 1	21%; 51%; 28%

Data are presented as n (%) or mean ± sd.

**Table 3:** **APOE and CSF biomarker baseline descriptives by syndrome diagnosis**

	**Subjective complaints**	**MCI**	**Dementia**
APOE genotype			
APOE N	159	208	294
e4 noncarrier	96 (60%)	97 (47%)	137 (47%)
e4 heterozygous	51 (32%)	90 (43%)	124 (42%)
e4 homozygous	12 (8%)	19 (9%)	29 (10%)
CSF biomarkers			
CSF biomarker N	60	67	84
Amyloid-beta 1-42	817 ± 252	692 ± 290	610 ± 258
Total tau	286 ± 170	473 ± 257	514 ± 277
Ptau	38 ± 18	53 ± 26	57 ± 26

Data are presented as mean ± sd. Please note that while raw CSF biomarker values are presented, statistical analyses were performed using log-transformed values.

### Clinical data

Data were collected on demographics, medical history, medication use, and family history from an open interview with both patient and caregiver. In addition, a clinician performed a physical examination. Finally, clinical assessment included several scales and questionnaires, as well as a standardized cognitive assessment.

#### Demographic data

Demographic variables included age, gender, educational level, ethnicity, and marital status.

#### Physical examination

The physical examination included the measurement of blood pressure, weight, height, gait disturbances, and extrapyramidal symptoms. In addition, data on smoking behavior, and alcohol consumption habits were collected.

#### Syndromal diagnosis

The diagnosis of dementia was based on DSM-IV criteria [16]. Etiological diagnoses were made according to standardized clinical criteria for AD (NINCDS-ADRDA criteria, [16,17], vascular dementia (NINDS-AIREN criteria, [18], frontotemporal dementia [3], and Lewy body dementia [2].

#### Medical history and medication use

Data were collected on medical conditions, such as neurological problems, cardiovascular problems, cerebrovascular problems, endocrine diseases, psychiatric diseases, and somatic diseases. In addition, data on the current use of medication (name, dose, frequency, and Anatomical Therapeutic Chemical (ATC)-code) were collected.

#### Family history

Data were collected on the family history of diagnoses of dementia, Parkinsonism, and Amyotrophic Lateral Sclerosis, including the number of first and second-degree relatives, the specification of the relatives, and their age at time of diagnosis.

#### Scales and questionnaires

The CDR, rated by the physician, provided a global rating of dementia severity. The 15-item Geriatric Depression Scale (GDS-15) [19] and the Euro-Quality of life-5 Dimensions [20] were used to provide self-reported information about depressive symptoms and quality of life. The 4-item questionnaire on Subjective Cognitive Functioning (SCF) gave information about the patient’s experience of his change in cognitive functioning. A semi-structured interview with a familiar caregiver provided information about the presence of neuropsychiatric symptoms and the daily functioning. The frequency and severity of neuropsychiatric symptoms were measured using the Neuropsychiatric Inventory (NPI) [21]. Basic and instrumental activities in daily life were assessed with the Disability assessment for Dementia (DAD) [22].

#### Cognitive assessment

Data about the start and course of the cognitive complaints were collected. The cognitive assessment consisted of a standardized battery of cognitive tests. All tests were performed according to the SOP for cognitive assessment. The tests were harmonized across all centers. An overview of the tests used and their corresponding cognitive domains is presented in Box 1.

**Box 1:** **Standardized cognitive test battery**

**Test**	**Cognitive domain(s)**
Mini Mental State Examination^1^	Global cognition
15 Word-Auditory Verbal Learning Test^2^	Episodic memory
Visual Association Test, short version^3^	Implicit associative visual learning
Digit-Span of the WAIS III (forward and backward)^4^	Working memory
Fluency, 60 seconds (animals)^5^	Verbal word fluency/semantic memory
Letter Digit Substitution Test, 60 seconds^6^	Information processing speed
Stroop Color Word Test (SCWT)^7^, 10x10 items, 4 colors	Information processing speed, attention and respons inhibition/executive functioning
Trail Making Test (TMT) (Part A and B)^8^	Information processing speed, attention and concept shifting/executive functioning
Optional: dot counting and incomplete letters of the Visual Object and Space Perception Battery	Visual perception

References: 1 [15]; 2 [23,24]; 3 [25]; 4 [26]; 5 [27]; 6 [28]; 7 [29,30]; 8 [31].

### Biobank

#### MRI

A standardized MRI protocol was defined in the SOP for brain imaging. Box 2 gives an overview of the MRI sequences included in the protocol. Acquisition of the MR images was performed at each individual site using MR systems operating at 1.5 or 3.0 Tesla. For each individual site, scanning was performed using the same scanner and headcoil throughout the study. After acquisition, scans were anonymized according to predefined and centralized guidelines. Pseudonymised data were subsequently transferred to the Image Analysis Centre (IAC) of the VUmc in Amsterdam, the Netherlands. The IAC collected, reviewed and stored the MR images. As part of the quality control (e.g. being compared by the local dummy scan), each MRI was centrally read by a neuroradiologist or trained rater and rated according to a number of basic visual rating scales: Medial Temporal Lobe Atrophy (MTA) [32], Global Cortical Atrophy (GCA) [33], White Matter Hyperintensities (WMH) [34]. In addition, lacunar infarcts, cortical infarcts, and microbleeds were counted.

**Box 2:** **MRI protocol**

1.	**Sagittal 3D T1-weighted gradient-echo sequence (6–9 min).**
	E.g. MP-RAGE, SPGR, FFE
	Correction for non-linear gradients (if applicable)
	TE and TR according to local settings- giving good gray matter – white matter contrast
	Slab thickness 180 mm, 180 partitions, 1.0 mm effective slice thickness
	In-plane resolution 1.0 mm
2.	2D T2* gradient-echo sequence (3–5 min)
	e.g. FLASH/FFE/SPGR, or (EPI-) SWI to obtain dark tissue background susceptible to T2* contrast –such as microbleedings.
	TR ≥ 500 ms, TE ≥ 20 ms. Flip angle 20 degrees
	Slice thickness 3 mm, no gap
	Number of slices 48
	In-plane resolution 0.5 – 1.0 mm
3.	**2D T2-weighted FLAIR turbo/fast spin-echo (3–5 min)**
	TR 8,000 – 12,000 ms, TE 100–150 ms, TI 2,200 – 2,800 ms
	Slice thickness 3 mm, no gap
	Number of slices 48
	In-plane resolution 0.5 – 1.0 mm
4.	**2D T2-weighted turbo/fast spin-echo (3–5 min)**
	TR 2,000 - 4,000 ms, TE 80–120 ms
	Slice thickness 3 mm, no gap
	Number of slices 48
	In-plane resolution 0.5 -1.0 mm
5.	**2D Diffusion weighted imaging/EPI (30 sec-1 min)**
	Parallel Imaging on if possible (mandatory for 3 T)
	TR 3,000-6,000 ms, TE 90–130 ms
	Slice thickness < 5 mm, gap 10-30%
	In plane resolution 2 mm b value (0/500/1000) or b value (0/900) or b value (0/1000) s/mm^2^.
	5b. Include online calculated ADC maps if possible.
6.	**2D Diffusion tensor imaging** (optional) (6–8 min)
	Parallel Imaging on if possible (mandatory for 3 T)
	TR 8,000-14,000 ms, TE 80–100 ms
	Number of slices 56–70 slices
	In-plane resolution 2.0 – 2.5 mm.
	30–60 diffusion weighted directions with b = 1000 s/mm^2^ , 5–6 scans with b = 0 s/mm^2^
	Fat suppression ON
7.	**2D Resting state fMRI** (optional) (7–10 min)
	**3.0 Tesla scanner:**
	TR 1,800-2,200 ms, TE 30–40 ms, Flip Angle 80 degrees
	Slice thickness 2.0 – 2.5 mm, gap 10%
	Number of slices 38–54
	In-plane resolution 2.0 – 2.3 mm
	Number of volumes 200 (excluding run-in scans)
	**1.5 Tesla scanner:**
	TR 2,200-3,000 ms, TE 40–60 ms. Flip Angle 90 degrees
	Slice thickness 2.5-3.0 mm, gap 10%
	Number of slices 30–38
	In-plane resolution 2.5 – 3.3 mm
	Number of volumes 200 (excluding run-in scans)

All scans have full brain coverage except DTI, which has coverage of at least 140 mm and resting state fMRI that had coverage of at least 105 mm. All scans were in the transverse orientation except the 3D T1w gradient echo sequence, which was in the sagittal orientation.

#### Blood

Samples of venous blood (10 cc clotted blood for serum and 6 cc EDTA blood for plasma) were collected. Samples were aliquoted into 0.5 cc samples and stored at −80°C in each local biobank.

#### DNA

6 cc EDTA whole blood was collected for DNA extraction. DNA was isolated at each individual site. DNA isolation was performed robotically, based on a salting out method. After isolation and a quality control, the DNA was stored in four cups at −80°C. Isolated DNA was taken from the local biobanks and transported to the department of Clinical Genetics of the Maastricht University Medical Center. Apolipoprotein E (APOE) genotype was determined on genomic DNA using the polymerase chain reaction (PCR) technique [35]. Genotyping was done blinded for all clinical data.

#### CSF

CSF was collected via a lumbar puncture in the intervertebral space at level L3/L4 or L4/L5. CSF was collected and stored in polypropylene tubes. Three cc of CSF was aliquoted in 0.5 cc samples and stored at −80°C until analysis.

CSF samples were transported to the department of Clinical Chemistry of the VUmc Amsterdam. The samples were analyzed all at once, using the same batch of reagents. Ab42, tau, and ptau concentrations were measured using commercially available single-parameter ELISA methods (respectively Innotest® beta-amyloid (1–42) and Innotest ® hTAU-Ag; Innogenetics, Ghent, Belgium). CSF analyses were done blinded for all clinical data.

### Follow-up assessment

One and two years after baseline, patients were invited for a follow-up assessment. At this time, the same clinical and cognitive data were obtained. In addition, the follow-up assessment two years after baseline also included the collection of blood samples and an MRI of the brain (Table 1). At follow-up, the course of cognitive problems was investigated and the syndromal and etiological diagnosis of each patient was based on the standardized criteria used at baseline.

Subjects who were not available for a follow-up assessment were contacted by telephone, using a standardized case record form (CRF) for telephone interview. This CRF included information on survival, the CDR, information about the reason for not participating in the follow-up assessment, the course of the cognitive complaints, interference with daily living, reasons for eventual hospital visitations, and whether a diagnosis of dementia was made since the previous measurement. Recently, the consortium has prolonged the clinical follow-up to at least three years, so the study is ongoing.

### Data collection, processing and storage

At baseline and follow-up, data are collected in a CRF based on SOPs. Data of the CRFs are locally stored in each center, based on a centrally defined Process Information Model (PIM) (www.string-of-pearls.org). The use of a PIM guarantees harmonized data collection across UMCs, and across diseases for shared variables, like demographics and medication use. After pseudonymisation, data were uploaded to a central data system. At a central level, PSI facilitates collaborative research and delivers a standardized Central Infrastructure, in which all clinical data collected by the UMCs are managed at one location.

Biological samples were processed and stored in each local biobank according to the SOP for biomaterials. For standardized analysis, biological samples were transported on dry ice to central laboratories according to the SOP for the transportation of biomaterials.

### Ethical considerations

The Medical Ethics Review Committee of the VU University Medical Center performed central approval of the study. All local Medical Ethical Committees approved the local performance of the study. The research is performed according to the principles of the Declaration of Helsinki (October 2008, www.wma.net) and in accordance with the Medical Research Involving Human Subjects Act and codes on ‘good use’ of clinical data and biological samples as developed by the Dutch Federation of Medical Scientific Societies. In addition, PSI has provided a regulatory framework in which ethical and legal rules and guidelines have been described. For guaranteeing the privacy of the patients’ data and biomaterials, a pseudonymisation service is delivered by a Trusted Third Party. All patients gave written informed consent.

## Results

### Preliminary baseline results

In total more than 1000 patients have been included, of which of a subsample of 665 patients, data were available for the present analysis. Data from this subsample of patients were at present available and could be extracted from the local UMCs and combined in one dataset, according to the regulatory framework of PSI. Slightly less than half of the patients were female. Average age was 70 ± 10 years old (Table 2). MMSE and CDR values show that in the majority of patients cognition was mildly impaired (Table 2). In line with this, the syndrome diagnosis was distributed as follows: subjective memory complaints (24%), MCI (31%), and dementia (45%).

APOE genotype measurement was available for 661 (99%) patients and CSF biomarkers for 211 (32%) patients. Table 3 shows that patients with dementia were more likely to be APOE e4 positive than patients with subjective complaints, with patients with MCI in between. Mean CSF concentrations of ab42 were lower while CSF concentrations of (p)tau were higher in dementia patients than in patients with subjective complaints. For all CSF biomarkers, the mean concentrations of MCI were intermediate.

## Discussion

The Pearl Neurodegenerative Diseases is a nationwide network enabling centralized and uniform collection of clinical data, blood, CSF and MRI of the brain in patients from tertiary memory clinics. To date, this has resulted in harmonization of data collection across centers and in the inclusion of more than 1000 patients with cognitive complaints, MCI or dementia. The current preliminary biomarker study has shown feasibility of collecting biomaterial from local biobanks for central analysis and coupling with phenotypical data, showing the potential of our data set.

The infrastructure of the Pearl Neurodegenerative Diseases, taking advantage of the PSI Central Infrastructure and regulatory framework, provides an excellent starting point for future research, including collaborations with other (inter)national projects focusing on neurodegenerative diseases. The dataset makes it possible to perform nationwide research into early diagnosis of neurodegenerative diseases, in particular AD. The uniqueness of this data set lies in the national coverage as our consortium includes all Dutch UMCs. Among the most important achievements until now is the harmonization of diagnostic procedures across academic memory centers. The use of SOP's and therefore a strict uniform data collection guarantees high quality of the data. We already see that other local memory clinics start to adopt the PSI-procedures, resulting in further standardization of diagnostics across the country and opening up further possibilities for collaborating in research projects.

The infrastructure of the Pearl Neurodegenerative Diseases is used as a framework for other studies, including the Dutch LeARN AD study [36]. In addition, both clinical data and biomaterial (specifically CSF) have already been incorporated in large international consortia (BIOMARK-APD; EMIF-AD) in the context of the Joint Programming of Neurodegenerative Diseases (JPND; www.neurodegenerationresearch.eu) and Innovative Medicines Initiative (IMI; www.imi.europa.eu) of the European Union.

Several other multicentre initiatives have collected data to study the value of new biomarkers for early identification of AD and related disorders. The Alzheimer’s Disease Neuroimaging Initiative (ADNI) in North America aims to identify neuroimaging measures and biomarkers associated with cognitive and functional changes in healthy elderly subjects and in subjects who have MCI and AD (www.adni-info.org). The Australian Imaging, Biomarkers and Lifestyle (AIBL) study of ageing aims to study biomarkers, cognitive characteristics and health and lifestyle factors determine AD (www.aibl.csiro.au). The Swedish Brain Power Network (www.swedishbrainpower.se) aims to enhance early diagnosis, treatment and care for patients with neurodegenerative diseases, including AD, Parkinson's Disease and amyotrophic lateral sclerosis. The Pearl Neurodegenerative Diseases adds to these other programs by firmly consolidating research in a clinical setting. The implementation of SOPs in clinical routine guarantees maximally efficient use of data and is thought to allow swift feedback of research results into clinical practice.

Among the limitations of the Pearl Neurodegenerative diseases is the potential referral bias as a consequence of inclusion of patients at tertiary UMC. The study results are generalizable to a population of patients referred to an academic memory clinic, but not to patients referred to a general practitioner, or any other community hospital or from the general population. Our sample provides a good reflection however of the patient population for which biomarkers might have high relevance. In addition, we included a broad range of patients, covering the continuum of cognitive decline from subjective complaints until the clinical diagnosis of dementia, but not healthy controls. One might argue, however, that for the aims of the present Pearl Neurodegenerative Diseases that the comparison of patients with dementia with patients with subjective complaints has more clinical relevance than comparing patients with dementia to healthy controls.

Based on our recent experience, we have continued patient inclusion to enlarge our data set. In addition, the consortium has decided to prolong clinical follow-up to at least three years, to allow the study of biomarkers in relation to long-term follow-up.

## Conclusion

In conclusion, the deep phenotyping in combination with longitudinal data collection, the multi-center set up, and the firm basis in regular patient care, resulting in a sample of real life patients, rather than a highly selected research sample, make our sample attractive for future studies aiming to develop diagnostic biomarkers, prognostic biomarkers, ‘theranostic’ (i.e. predicting response to therapy) biomarkers and biomarkers aiming to monitor disease progression. As such, the Pearl Neurodegenerative Diseases forms an ideal real life sample to host studies aiming to improve trial design and to validate new targets of therapy.
